# Executive Functions and the Improvement of Thinking Abilities: The Intervention in Reading Comprehension

**DOI:** 10.3389/fpsyg.2016.00058

**Published:** 2016-02-04

**Authors:** Juan A. García-Madruga, Isabel Gómez-Veiga, José Ó. Vila

**Affiliations:** Psicología Evolutiva y de la Educación, Universidad Nacional de Educación a DistanciaMadrid, Spain

**Keywords:** executive functions, working memory, reading comprehension, Intervention programs, education

## Abstract

In this paper, we propose a preliminary theory of executive functions that address in a specific way their relationship with working memory (WM) and higher-level cognition. It includes: (a) four core on-line WM executive functions that are involved in every novel and complex cognitive task; (b) two higher order off-line executive functions, planning and revision, that are required to resolving the most complex intellectual abilities; and (c) emotional control that is involved in any complex, novel and difficult task. The main assumption is that efficiency on thinking abilities may be improved by specific instruction or training on the executive functions necessary to solving novel and complex tasks involved in these abilities. Evidence for the impact of our training proposal on WM's executive functions involved in higher-level cognitive abilities comes from three studies applying an adaptive program designed to improve reading comprehension in primary school students by boosting the core WM's executive functions involved in it: focusing on relevant information, switching (or shifting) between representations or tasks, connecting incoming information from text with long-term representations, updating of the semantic representation of the text in WM, and inhibition of irrelevant information. The results are consistent with the assumption that cognitive enhancements from the training intervention may have affected not only a specific but also a more domain-general mechanism involved in various executive functions. We discuss some methodological issues in the studies of effects of WM training on reading comprehension. The perspectives and limitations of our approach are finally discussed.

## Introduction

Human thought involves the building of mental representations by integrating external and previously stored information, and their manipulation in a cognitive space: working memory (WM). Thinking can involve a goal or it may just involve a wandering mind, but it always requires WM's activation and use. For this reason it is affected by its processing and storage limits. Higher-level thinking abilities such as complex text comprehension, deductive reasoning, writing, and meaningful school learning operate sequentially. They consist of diverse component subtasks and demand that people keep their attention focused throughout the entire process. Besides the initial construction of representations, higher cognitive tasks require individuals to keep the goal of the task in mind, to shift from one sub-task to the next, and to update representations by activating Long Term Memory (LTM) information. The fulfillment of these complex cognitive tasks demands people to activate all their WM resources in a controlled and supervised way. The more complex and novel the intellectual task an individual is faced with the more involved WM's executive processes are in its resolution.

There is an obvious corollary to the tight relationship between WM's executive processes and thinking abilities: One way to improve these abilities is by training people in the use and activation of executive processes during the execution of novel and complex tasks involved in these abilities. This approach has three main theoretical components: (a) a proposal about the executive processes involved in higher cognitive abilities; (b) an analysis of how these executive processes operate while carrying out the complex and novel tasks selected; and (c) a proposal regarding how the executive processes can be trained. The two first components are peculiar to our proposal; the third component is common with the current theoretical and experimental approach of WM training.

In this paper we shall present our theoretical approach and how it can be applied to the acquisition of reading comprehension in childhood. In the next section we will address the relationship between WM and executive functions, and explain our theoretical proposal regarding executive functions and its development. After that, we will tackle a central issue for training: the modifiability of WM and executive functions. Later we will describe our training program on WM's executive functions involved in reading comprehension. The improvement of reading comprehension in primary school using our approach has been confirmed already and we will include some of the results we have found in diverse experiments. Finally, we will address the perspectives and limitations of our theoretical conception on the improvement of thinking abilities.

## Working memory and executive functions

Working memory and executive functions (EFs) are tightly related but have diverse theoretical and experimental origins. EFs have its origin in neuropsychology, particularly in the work of Alexander Luria. Although the term “executive function” comes from Lezak (1982), Luria was the first author who conceptualized it. Luria (1966) was a prominent soviet neuropsychologist whose work led him to postulate connections among the frontal lobes (or the prefrontal cortices, PFC), executive functioning and problem solving. He documented the behaviors of individuals who suffered frontal lobe damage while they attempted to solve a problem and concluded that problem-solving behavior was dependent on a number of essential skills, or executive functions, which were dependent on the frontal lobes. Luria described the main components of executive functioning as: anticipation (setting realistic expectations, understanding consequences), planning (organization), execution (flexibility, maintaining set), and self-monitoring (emotional control, error recognition).

During the last quarter of the past century extensive work in this area has been done. Different functional circuits within the prefrontal cortex have been described from a neuroanatomical point of view. This work has confirmed the role of the frontal lobes in executive functioning (Fuster, 1989; Cummings, 1993). The idea that every executive process is mediated by the PFC (i.e., the frontal executive hypothesis), has long been widely accepted. It provides a conceptual framework for the belief that all executive processes are alike in critical ways. However, diverse studies have shown that EFs do not depend solely on the PFC. Other cortical and non-cortical regions of the brain are also involved in the cognitive and emotional processes that we call EFs (see Alvarez and Emory, 2004).

The development of diverse brain regions directly related with EFs, particularly the PFC but also the anterior cingulate, parietal cortex, and the hippocampus, is particularly relevant in infancy and early childhood. This development entails the early overproduction of synaptic connections, followed by their selective pruning or reduction, and the establishment of new circuits and interconnections between diverse brain regions (see Johnson, 1998; Diamond, 2002). Whereas maturation and structural development seem to predominate in childhood, an important development of efficiency in the use of available cognitive resources occurs from 10 to 12 years that results in large performance differences between children and young people (e.g., Jolles et al., 2011).

As a matter of fact, the study of brain development has shown that adolescence is a critical stage. A number of studies have shown that in this age period structural changes are still occurring in the prefrontal cortex: the proliferation of synapses occurs up to adolescence, and the pruning of neuronal connections continues till the third decade of life in adulthood (Blakemore and Frith, 2005). However, the most relevant modification in the adolescent brain is likely the global increase in the myelinization process. The enlargement of the sheath that covers and isolates neuronal axons is responsible for an increase in the speed of neural connection. It thus yields a parallel rise of the efficiency of brain computations underpinning the development of intellectual abilities and particularly EFs (Nelson et al., 2006).

In spite of extensive work carried out during the past 30 years and the increase in neuroscientific evidence regarding their cortical underpinning, EFs are still considered an elusive concept (Jurado and Rosselli, 2007) that possibly involves some confusion (Klenberg et al., 2001). Most of the confusion comes from their tight relationship with WM and with higher-level cognitive abilities. In order to reduce this conceptual confusion, it is necessary to clarify the relationship between EFs and WM, as well as to distinguish EFs from higher thinking abilities such as comprehension, reasoning, or problem solving. In this paper, we propose a preliminary theory of EFs that address in a specific way their relationship with WM and higher-level cognition. This conception is mainly based on two influential perspectives: Diamond's cognitive developmental neuroscience work on EFs, and experimental work on executive control processes in the fields of attention and WM.

Diamond (2009, 2013) has developed one of the most comprehensive proposals on the EFs. According to Diamond, the EFs enable the mental manipulation of ideas, thinking before acting, managing novel information and unanticipated challenges, inhibiting and resisting temptations, and staying focused during the execution of difficult tasks. Diamond notes there is general agreement that there are three core EFs: (1) working memory that holds information in mind and works with it; (2) inhibition or inhibitory control, including self-control (behavioral inhibition), and interference control (selective attention and cognitive inhibition); and (3) cognitive flexibility, that is adapting cognitive behavior to changing demands or priorities. Cognitive flexibility is related to task switching and is the opposite of rigidity. According to Diamond, there are other higher order EFs, such as thinking, problem solving, and reasoning, which are built from the core EFs (Collins and Koechlin, 2012; Lunt et al., 2012). As we will see, our proposal shares with Diamond the existence of core and higher order EFs. However, our view regarding the relation between WM and EFs, as well as between EFs and thinking abilities, differs from Diamond's view.

A second crucial perspective on the EFs comes from experimental research on cognition, particularly attention, and WM. Attention can be defined as the prioritization of information matching the individual's task goals (Nobre and Stokes, 2011). Attention has been treated as representing a cognitive filter (Broadbent, 1958), a basic model to stimuli orientation (Posner, 1980), but also as a control process of WM (Shiffrin and Schneider, 1977). Attention and WM are tightly related. In fact, they are increasingly viewed as overlapping constructs (see Awh et al., 2006; Gazzaley and Nobre, 2011). Thus, recent theoretical models of WM describe a function for attention, although in these models there is not much agreement on its specific role.

Within this perspective, the work by Miyake et al. (2000) has been particularly influential (see Garon et al., 2008). These authors carried out a differential study with university students that found support for the existence of three main EFs: (1) response inhibition (the ability to inhibit dominant, automatic, or pre-potent responses), (2) updating WM representations (the ability to monitor incoming information for relevance to the task at hand and then appropriately update it by replacing older, no longer relevant information with newer, more relevant information), and (3) set shifting (the ability to flexibly switch back and forth between tasks or mental sets). These authors showed that these three EFs are diverse, but tightly interrelated and overlapping. Recent neuroimaging studies also indicate unity and diversity of EFs in terms of brain localization (Collette et al., 2005). Likewise, a number of authors have addressed the question of whether the unity/diversity framework appropriately describes the structure of EFs in children, adolescents and adults (see, e.g., Miyake and Friedman, 2012). Findings indicate that the latent factor structure of executive control changes qualitatively over development, from a unitary structure in preschoolers to multiple components in school-age children and adolescents.

The study of the relationship between EFs and higher-level cognition has been frequently carried out according to Baddeley's multiple-component model of WM (Baddeley and Hitch, 1974; Baddeley, 1986, 2000). According to this theory, the WM system includes two domain-specific storage structures or slave systems (the phonological loop and the visuo-spatial sketchpad), an episodic buffer that links these two components with LTM, and a central executive (CE). The CE is the main component of the WM system and it is in essence based on the “supervisory attention system” described by Norman and Shallice (1986). CE not only has to coordinate the other components but is also in charge of the attentional control of information. Two related and influential models of WM are: Cowan's (1999) embedded-processes model, and Engle's (2001; Unsworth and Engle, 2007) general capacity model. In spite of their differences, Baddeley's, Cowan's, and Engle's models all share the idea of a domain-general CE in charge of controlling cognitive resources while solving new or difficult tasks.

Following the recent proposals of these authors (see Miyake et al., 2000; Engle, 2002; Cowan, 2005; Baddeley, 2007), in this paper we claim that there are four main core WM EFs involved in the on-line execution and monitoring of complex intellectual tasks: to focus and sustain attention, to switch attention, to activate and update representations, and to inhibit automatic processes and discard irrelevant information. All four of these CE processes demand cognitive effort and resources.

Focusing and sustaining attention is an EF that is required in order to solve any non-automated task. It involves the capacity to resist possible distractors and keep attention focused on a task. During infancy, focusing may be a difficult task since at this age attention is mainly determined by environmental factors such as novelty. In complex tasks, focusing allows individuals to orient their attention on a number of elements or blocks of information, keeping them in their mental space in a voluntary and conscious way.

A second CE function, related with focusing, is the capacity to switch attention. It allows changing one's attention from one stimuli, representation, or process to another, according to internal goals and task demands. In order to solve complex tasks, we must not only be able to fix our attention on those elements (stimuli or representations), or processes relevant to the execution of a task, but also must be able to shift our attention to other necessary aspects or components of the task. Thus, switching attention involves moving in a flexible way the focus of attention from one entity to another. This is supported by meta-analytical neuroimaging studies that provide neural evidence for switching (Wager and Smith, 2003). These studies conclude that switching seems to involve neural mechanisms located in the parietal cortex, which again argues against the exclusive frontal-executive hypothesis.

Processes related to “updating” information not only involve the simple active maintenance of relevant ongoing processing elements, but they also involve a “review” of the “fitness” of the representations generated and managed from new elements (i.e., a kind of “supervision” and “monitoring” of the information when approaching the objectives of the task). This is what happens for example during text comprehension. The process of text understanding requires readers to activate prior knowledge in order to continuously achieve appropriate semantic synthesis, and thus to update mental representations regarding the meaning of the text. Recent research confirming the capacity of updating to predict fluid intelligence explicitly stresses the importance of this process in higher-level cognition (see Friedman et al., 2006; Chen and Li, 2007; Belachi et al., 2010).

The capacity to inhibit information or representations that are not relevant to the task involves prioritizing the processing of some types of information over other types. However, inhibition involves not only the process of selecting information, but also the capacity to resist new information while maintaining essential information relevant to carry out the online task (see Borella et al., 2008). Thus, the inhibitory control of attention enables us to selectively attend, focusing on what we choose to attend to and suppressing any other stimuli, processes, or responses. These processes are critical in those complex tasks in which information processing are beyond the capabilities of the WM. A way to avoid overloading WM is to inhibit irrelevant representations and discard unnecessary information. Another ability related to inhibition is cognitive reflection (see Frederick, 2005). This ability involves controlling the behavior in a thoughtful way and inhibiting the first answer that comes “to mind” when solving difficult intellectual problems.

As we can see, our view includes the three classic executive functions proposed and tested in a classical study by Miyake et al. (2000): shifting, updating and inhibition, with the addition of a fourth component: focusing. The relevance of focusing is widely recognized in education: as every teacher knows, focusing and sustaining attention is a main executive process in school learning. In fact, according to Baddeley (2007), the capacity to focus and direct attention is probably WM's most crucial EF. One influential perspective on the role of WM capacity in mental work and cognitive development makes a different claim (see Pascual-Leone, 1987, 2000). It argues for the relevance of a component of mental attention that allows one to allocate capacity-limited attention to representations held in WM. According to Pascual-Leone, mental-attentional capacity is a limited capacity to hold in mind at any one time different information elements or schemes that are relevant for intellectual task resolution. Mental capacity is counteracted by a mechanism of mental attention interruption that corresponds to the ability to actively interrupt or inhibit the schemes that are not relevant to the task. From this perspective, Im-Bolter et al. (2006) proposed a fourth components model. Besides the two basic attentional components, mental activation capacity and mental inhibition capacity, they also include two executive components: shifting and updating. This model has shown its predictive capacity in children with specific language impairment. It has been extended to the study of how these four components contribute to children's ability to solve multiplication word problems (Agostino et al., 2010). The main difference between this model and the proposal of this paper is that we consider the four components as central EFs of WM.

When resolving a complex and novel task, such as reading a difficult text, the actions of these four WM executive functions are tightly related. Resolving a task as such always requires breaking it down into subtasks, and thus focusing and switching attention between these. It also demands that individuals retrieve knowledge stored in LTM in order to update representations during the execution process. This updating, however, also implies inhibiting older elements and information in its representation. Likewise, in order to be able to inhibit representations and discard information, individuals have to be focused on the relevant components of the task and resist and sustain their attentional focus in spite of the temptations invoked by the context or the stimuli itself.

Although, our main objective in this paper is centered on the four core EFs that concern the present on-line control and resolution of a cognitive task, our conception, following Diamond's proposal, entails also other higher order EFs (see Table 1). There are EFs that focus not only on on-line tasks, but instead on the future (i.e., planning), or on past behavior (i.e., revision). These higher order EFs are required in most complex cognitive abilities such as problem solving, reasoning, and writing. Planning involves the selection, formulation and evaluation of a sequence of thoughts and actions to achieve a desired goal (see Morris and Ward, 2005). It allows a person to analyze and adjust the available information, as well as the strategies and processes needed to solve tasks. In fact, problem solving and planning tasks, such as the Tower of Hanoi or the Tower of London, have frequently been used to measure executive functioning especially sensitive to frontal lobes dysfunction (see Goel and Grafman, 1995).

**Table 1:** **Main types of executive functions**.

**General Characteristics**	**Executive Functions**
WM's on-line core EFs Every complex and novel cognitive task demand their use	*Focusing and sustaining attention Switching attention* *Activating and updating representations Inhibition of responses and information*
Off-line higher order EFs Most complex intellectual abilities such as reasoning and problem solving demand their use. They are carried out within WM and require to apply core WM's EFs	*Planning future behavior*
	*Revision of task execution*
Emotional processes They are involved in solving any kind of complex, novel and difficult task.	*Emotional control of behavior*

Likewise, the ability to successfully resolve complex thinking tasks is associated with the need to evaluate the processes and results that make up the diverse tasks executed during its resolution. Thus, a final revision mechanism is needed to ensure that actions are performed in line with prior demands. Writing is probably the clearest example of complex intellectual ability that requires revision (Allal et al., 2004). Other examples of this need for revision are: solving mathematical problems or drawing deductive reasoning inferences, but also processes relating to complete understanding and learning about complex matters. Revision should be focused on the analysis and control of procedures applied and implemented to ensure a correct resolution.

Apart from the core and higher order cognitive EFs, there is another executive function clearly involved in an individual's action: the emotional control of behavior. In other words, the ability to modulate emotional responses by bringing rational thought to bear on (or resist) our own feelings. Emotional control underlies all human behavior, including higher-level cognition and the executive processes previously analyzed and described. In fact, emotional activation can interfere with cognitive control processes in healthy individuals, and thus depression is associated with impaired disengagement from negative information (Aker and Landro, 2014).

Therefore, our theoretical proposal claims the existence of three main kinds of EFs: (a) on-line core WM executive functions: focusing attention, switching attention, activating and updating representations, and the inhibition of automatic processes and responses; (b) off-line higher order EFs centered either on planning future cognitive behavior or on revising prior behavior already executed; and (c) emotional control that includes not only the control of desires and affections, but also the control of anxiety and emotions that underlie the execution of new and complex intellectual activities.

Higher order EFs, planning, and revising, involve the four core EFs since they are also carried out within WM, even if they are not necessary in some tasks and abilities, such as ordinary reading comprehension. As we can see, all cognitive EFs are tightly related with WM: the four core EFs are part of the CE functions; and the two higher EFs are the result of applying core EFs to the task of foreseeing and organizing future actions, and to reviewing and evaluating prior behavior and actions. Another difference between core and higher order EFs is that the latter overload WM and frequently require external support or memory. The difference between core and higher order EFs is also clearly shown in their development.

As diverse authors have shown, the first years of life are crucial in the development of core EFs (Diamond, 2006; Garon et al., 2008). For example, a rudimentary ability to select a stimulus and to focus attention is present early in infancy. The development of attention during infancy allows preschoolers to focus on internal representation and resist the attraction of environmental stimuli (Rothbart and Posner, 2001). Focusing and shifting are obviously related but they seem to show separate developmental paths in early infancy (see Posner et al., 2006). The ability to shift attention between two objects appears during the 1st year, and in the 2nd year children should already be able to shift between an internal representation and a perceived stimulus. Likewise, from 3 to 5 years old, children show a significant improvement in attention switching between tasks when the active maintenance of information and inhibition is required (Diamond, 2002). There are diverse response inhibition tasks that can be labeled as simple and complex (see Garon et al., 2008). Simple inhibition tasks, such as the ability to suppress a dominant response, involve a minimal WM demand, and they develop in the 1st year of life. Complex inhibition tasks involve a higher WM demand, such as in Stroop tasks that require people to hold a verbal rule in mind, respond according to it, and inhibit an automatic response. The development and acquisition of this kind of complex inhibition comes later, from 3 to 5 years old (see Garon et al., 2008). The study of updating by requiring participants to recall the last items of a list of letters was first used by Morris and Jones (1990), however there is very little evidence of its development. Belachi et al. (2010) used a more complex relevance-based updating task (see Palladino et al., 2001), in which participants were asked to remember the smallest items of a list of objects. They found a linear pattern that increased with age in children between 5 and 11 years old, similar to that obtained with other measures of WM and fluid intelligence. As we see below, we used a semantic updating task in two studies.

The core EFs, as different studies have shown (e.g., Huizinga et al., 2006; Best et al., 2009), continue to develop until adolescence or even young adulthood. The study of the development of higher order EFs, planning and revision, is practically inexistent and there is little known about it (see however, Nurmi, 1991). Although planning and revising also begins to develop in infancy, higher order EFs are of belated acquisition. The age period when they mainly develop and reach their maximum level is late adolescence and young adulthood. In a parallel way and underlying the development of most complex thinking abilities, the development of higher order EFs is likely result of the multiple and repeated realization of diverse complex intellectual tasks in educational contexts (see Best et al., 2009).

## The modifiability and training of WM and EFs

A number of studies focused on training-induced cognitive and neural plasticity have provided evidence that cognitive abilities and brain activity are potentially modifiable (see e.g., Karbach and Schubert, 2013). Consistent with this view, many studies have investigated the effectiveness of cognitive training interventions to improve WM, as well as to help overcome cognitive deficits or learning difficulties (for reviews, see Morrison and Chein, 2011; Shipstead et al., 2012; Titz and Karbach, 2014; von Bastian and Oberauer, 2014). Growing empirical evidence indicates that WM training interventions can lead to real and lasting gains not only in typically developing pre-schoolers (see Diamond, 2012), in school-aged children and adolescents (for a review, see Karbach and Unger, 2014) up to adulthood (e.g., Karbach and Kray, 2009), but also in children with cognitive deficits or learning difficulties (Klingberg, 2010). This is true particularly for studies investigating the benefits of WM training programs that involve adaptive tasks (i.e., tasks in which participants are given many trials to perform that are at or slightly above their current ability). The meta-analytic review undertaken by Melby-Lervâg and Hulme (2013)—including studies with clinical and typically developing samples of children and adults—indicates that WM training programs produce significant and immediate improvements in measures of verbal WM, with larger gains occurring in studies with younger children (below age 10 years) relative to older children, as well as moderately sized immediate gains on measures of visuospatial WM. These authors conclude that, even though memory training programs appear to produce short-term specific training effects, there is no clear evidence that such benefits are durable and generalizable to other skills.

It may be noted that one aim in many WM training interventions is not only to improve performance on WM tasks, but also to obtain transfer or generalizing effects to new tasks or domains that have not been trained (for a discussion, see von Bastian and Oberauer, 2014). Theoretically, if it is assumed that WM reflects a general attentional resource limitation, and considering the strong relation between WM and performance in a multitude of tasks (Cowan, 2005), we would predict that training WM, if successful, should show transfer effects to untrained tasks (Shipstead et al., 2012). The underlying idea is that training should lead to an increase in a domain-general attentional capacity that is critical for performing many diverse tasks. Particularly, the improvements in WM functions might be beneficial for individuals with poor WM skills and for those who are at risk of learning difficulties (e.g., Gathercole and Alloway, 2008). It, therefore, appears necessary to assess the extent to which WM training programs are effective in increasing measures on tasks similar to those trained (near-transfer effects), as well as on scores on tasks that have not been trained directly (far-transfer effects), either within the same cognitive domain or even to more general cognitive abilities relying on WM. In that respect, a number of recent studies provide some evidence that WM training can optimize an individual's performance in a number of other cognitive measures. For instance, Klingberg et al. (2002) reported that young adults trained using a protocol that combines multiple WM tasks improved significantly on cognitive control and general fluid intelligence measures. Using the same paradigm, these authors also found similar improvements in cognitive control and general fluid intelligence in children with ADHD (see also Klingberg et al., 2005).

As for executive processes, there are very few studies that have specifically investigated transfer from WM training to EFs. For instance, Salminen et al. (2012) investigated transfer effects from WM training to different aspects of executive functioning. Participants were trained on an adaptive complex task that requires simultaneous performance of a visual and an auditory *n*-back task. Transfer tasks measured four executive processes separately: WM updating, coordinating the performance of simultaneous tasks (dual task) and sequential tasks (task switching), and the temporality of attentional processing. The results indicate that, following training, participants improved in the trained task, in the WM updating transfer task, in a task switching situation, and in attentional processing. However, there was no transfer to the dual task. Further evidence comes from other studies showing that training on task-switching improves cognitive flexibility and generalizes to new untrained tasks assessing other dimensions of executive functioning (e.g., Karbach and Kray, 2009).

However, the conclusions about transfer effects from WM training are not consistent across studies, a fact that has stimulated a debate regarding the potential efficacy of training for improving not only WM but also related cognitive abilities (e.g., Titz and Karbach, 2014). The empirical evidence on the generalizability of training gains is quite mixed (for a discussion, see von Bastian and Oberauer, 2014). Some researchers indeed report only significant improvement on the trained tasks (e.g., Jaeggi et al., 2011). Others reveal occasional near transfer to tasks that were not explicitly trained but share similar task features with the training tasks (e.g., Dunning and Holmes, 2014), and sometimes even more far-removed transfer to tasks measuring a different construct (reading comprehension, e.g., (Dahlin, 2011); mathematics, e.g., (Holmes and Gathercole, 2014); fluid intelligence, e.g., Borella et al., 2010). Furthermore, the results of some studies show the maintenance of these effects (e.g., Dahlin, 2011), alongside others reporting that the effects were not maintained at follow-up measurements (St. Clair-Thompson et al., 2010). To date, it does not seem feasible to reject one of the positions in favor of the other. The inconsistency of results regarding the efficacy of WM training are explained by large differences in terms of the methodologies that have been adopted across studies (see Shipstead et al., 2012; Melby-Lervâg and Hulme, 2013). Besides methodological issues, to draw consistent conclusions about the effectiveness of WM training it is also important to consider that the magnitude of training-induced gains are potentially influenced by the underlying mechanisms mediating transfer, not to mention additional factors that could influence the success of training interventions (for a review, see von Bastian and Oberauer, 2014). Therefore, it seems more appropriate to analyze under which circumstances WM training can improve cognitive performance. Moreover, it remains open which type of training most efficiently supports the occurrence of transfer effects.

## Improving reading comprehension by training the involved WM's executive functions

We aim to contribute to the debate on the feasibility of WM executive functioning training. A relevant question we are concerned with is whether and to what extent interventions that contribute to enhancing WM's executive processes involved in higher-level cognitive abilities, such as reading comprehension, would improve these abilities. This is of particular relevance in childhood and adolescence, given that executive functioning is not only related to higher-level cognitive abilities contributing to academic success, but also to performance in the classroom (for reviews, see Swanson and Alloway, 2012; Titz and Karbach, 2014). In this section, we begin with an examination of studies demonstrating alternative approaches to WM training that provided evidence favoring the conclusion that WM training can benefit reading comprehension. We follow with a description of our training proposal on WM's executive functions involved in reading comprehension.

Reading comprehension is considered a complex and highly demanding cognitive task that involves the simultaneous process of extracting and constructing meaning (e.g., Kintsch, 1998). The functional role of WM in reading comprehension and its component skills has been well-established, both in typical developing children (Cain et al., 2004) and in individuals with poor reading comprehension abilities (e.g., Carretti et al., 2009). As numerous authors have maintained, WM plays a crucial role in storing the intermediate and final products of a readers' s computations, as well as coordinating the processes of constructing and integrating a semantic representation from the text (e.g., Just and Carpenter, 1992; Ericsson and Kintsch, 1995). Besides the role of the phonological component of WM for reading comprehension, growing evidence supports the involvement of the diverse yet interrelated CE processes and underscores the importance of attentional control (see e.g., De Jong, 2006). For instance, Swanson et al. (2006) pointed out that the EF of coordinating cognitive operations is required to integrate information from text and LTM. Likewise, Palladino et al. (2001; see also Carretti et al., 2005) linked WM's updating to reading comprehension skills. Also, whereas Yeniad et al. (2013) reported the relation between shifting and reading, De Beni and Palladino (2000) have underscored the function of inhibiting possible representations and discarding information in reading comprehension.

Despite the strong relation between WM's executive functions and reading comprehension, there are very few studies that have assessed the effects of WM and EFs training on reading comprehension. In that respect, a review by Titz and Karbach (2014) showed limited but converging evidence for positive effects of process-based complex WM training (i.e., training of specific cognitive processes, without explicit strategy training) on academic abilities, particularly in the domain of reading. The benefits were found in typically developed students as well as in children with cognitive deficits and learning difficulties. In contrast, other studies found significant improvements in tasks assessing the CE components of WM after training, but no improvements were found on reading comprehension (e.g., St. Clair-Thompson et al., 2010). A possible explanation of these contradictory results about the effectiveness of WM and EFs training may stem from differences regarding the kinds of interventions employed in existing studies and the characteristics of the study sample. More specifically, variations across studies were identified in several factors that could influence the success of training interventions, such as the type of training procedure, the intensity, and duration of training, stepwise adjustment of task difficulty to individual performance during training, or the design of the control conditions. The following discusses some methodological issues in the studies of effects of WM training on reading comprehension.

A subset of previous training studies are focused on a training procedure that elicits practice on only a single task (or several variants of one type of task), and which allows the individual to analyze specific aspects or functions of WM. For instance, Chein and Morrison (2010) developed an adaptive training protocol that involved verbal and spatial adaptive versions of a complex WM span task that taxes several different processes, such as encoding, attention, and WM updating. After 4 weeks of intensive training, participants (mean age of 20 years) improved significantly more than non-active controls on measures of complex WM span as well as on complex reading comprehension tasks, as measured by a standard reading test (Cohen's *d* = 0.58). Since training improved different abilities, the authors inferred that the training task must have affected a domain-general mechanism responsible for attentional control processes.

Positive transfer to reading comprehension has also been reported in studies using WM training protocols based on a range of computer-based memory tasks. The most well-known program is Cogmed WM Training battery (CWMT), a battery of video-game-like tasks, each aimed at improving WM and executive control (Klenberg et al., 2001; for a controversy on CWMT, see Shipstead et al., 2012). The difficulty level of each task is adjusted for each trial to ensure that the individual is working at her or his personal limits. For instance, in Dahlin's intervention study (2011), primary school students (9–12 years) with special needs were trained daily by using tasks from the CWMT for 30–40 min over a period of 5 weeks in school settings. The computerized training program included both visuo-spatial and verbal working memory tasks, with a fixed number of trials (100) to be completed each day. The results showed that, compared to the passive control group of Klingberg et al. (2005), children improved on reading comprehension (Cohen's *d* = 0.88), but not on word decoding or orthographic verification experimental tests, and the benefit was maintained for 6 months.

Along this line, two studies involving typically developing children have yielded consistent results by applying a computerized WM training intervention based on complex WM tasks from the Braintwinster battery (Buschkuehl et al., 2008). First, Loosli et al. (2012) applied a brief (10 sessions over 2 weeks), adaptive computerized WM training program based on a complex WM span task from the battery to train children (9–11 years) to improve reading performance. These authors found that, compared to a passive control condition, the training intervention significantly enhanced experimental group performance on the trained WM task, but also on a standardized reading test (Cohen *d* = 0.20). Particularly, WM training had a smaller impact on single-word reading performance than on text comprehension tasks. Second, Karbach et al. (2015) found that 14 sessions of an adaptive WM training applying tasks from the Brain twister battery improved performance in elementary-school children (mean age = 8.3 years) on untrained WM tasks and on a standardized test of reading abilities. Moreover, transfer to untrained WM tasks was maintained over 3 months. The analysis of individual differences revealed compensatory effects with larger gains in children with lower WM and reading scores at pretest.

As we can see, the aforementioned studies report findings that support the view that WM has the potential to improve reading comprehension. Prior evidence mainly comes from studies that have applied a process-based WM training program based on intensive practice on memory tasks. The applied tasks do not only require storage, but also additional processing demands. Thus, these training programs might rightly be considered CE training. Given this approach, training often yields large improvements on the trained tasks, but it also results in transfer effects to reading comprehension. Additionally, the interventions often implemented an adaptive training procedure. In that respect, Karbach et al. (2015) demonstrated that adaptive WM trained resulted in larger training gains than non-adaptive low-level training on the same tasks (active control group), but the question is still open whether adaptivity really plays an important role for the effectiveness of WM training interventions (see, von Bastian and Oberauer, 2014).

Nevertheless, there are certain issues in these studies that should be acknowledged. First, one common practice has been to compare the performance of the training group to that of a non-active control group, but one that did not attend any intervention. In this way, it raises the question of what degree performance changes within the training group can be attributed to the training tasks instead of to the existence of an intervention *per se* (Shipstead et al., 2012). Another issue that arises when trying to draw conclusions about the effectiveness of these training approaches is that the evidence is only based on one reading comprehension measure. The interpretation of these findings may be problematic because generalization could be the result of idiosyncratic relationships between the trained and assessment tasks, and not because of any enhancement in the underlying ability thought to be measured by the assessment task itself. As Shipstead et al. (2012) pointed out, transfer effects of training should be demonstrated using a wider variety of tasks.

On the other hand, most of the previous training programs have involved individual training sessions that were not part of classroom activities. Also, prior training procedures were implemented by researchers under controlled and intensive conditions that cannot feasibly be achieved in non-research situations. As a consequence, it is not clear how the training of WM is applicable in educational settings or to whole classes (see Gathercole et al., 2006). Interestingly, it was found that adaptive WM training based on tasks from CWMT battery transferred to new untrained WM tasks, but not to basic word reading abilities in children (8–11 years old) with low WM ability (Holmes et al., 2009; Dunning and Holmes, 2014). In contrast, a similar training program administered by teachers to their own pupils (aged 9–11 years) with low academic abilities improved, compared to a passive control group, children's performance in English, as measured by means of a national standard assessment test (Holmes and Gathercole, 2014). These results suggest that WM training has the potential to transfer to academic abilities, even when conducted by teachers in real-life conditions in schools, with effect sizes (Cohen's *d* effect sizes range from 0.56 to 0.67) comparable to those reported in research studies.

From an applied point of view, the importance of having the teaching of WM and its processes embedded into the classroom curriculum is obvious. Adopting this perspective, some WM and EFs training interventions are based on teaching strategies that address EFs in classroom activities (e.g., Meltzer et al., 2007; Gaskins et al., 2007) Among the few studies that have attempted to enhance WM by means of a range of activities suitable for including in the school timetable and conducting in classroom, the one conducted by Carretti et al. (2014) deserves mention. They implemented a training procedure that combined a range of activities focusing on WM and on metacognitive reflection in reading comprehension. After training by teachers, the authors found medium to large positive effects on reading comprehension skills in primary school children (8–10 years), and the effects were maintained after 11 months. These findings highlight the relevance of integrating WM training into the classroom curriculum.

As for our theoretical view, we propose a novel approach regarding how EFs can be improved through training in educational settings (for details, see García-Madruga et al., 2013). This new training program was designed to improve reading comprehension in primary school students by boosting the executive processes of WM involved in it: focusing on relevant information, switching (or shifting) between representations or tasks, connecting incoming information from text with long-term representations, updating of the semantic representation of the text in WM, and inhibition of irrelevant information. A few training principles were assumed that, as Diamond pointed out (2013, p. 154), seem to hold for effective training: (1) executive functioning training appears to transfer; (2) EFs demand needs to be continually and incrementally increased; and (3) practice is key.

A main feature of this training program is that it was directly implemented into reading comprehension activities. However, the main focus of the training procedure was not to train reading comprehension itself, but to train the conscious control of the cognitive processes involved in it. For this purpose, a variety of reading comprehension tasks were used for training, of which four core WM EFs are particularly involved (see Table 2). The focusing function on specific and relevant information to resolve the task is present in all of them. The switching function is particularly required on the tasks in which readers have to shift back and forth between diverse pieces of information or when the task includes diverse subtasks. Connecting with long-term knowledge is particularly necessary when performing tasks that require combining information from the task with information from long-term memory. The updating function is present in those tasks that require monitoring and coding incoming information relevant to the tasks at hand and then appropriately revising the items held in WM and replacing older, no longer relevant information with newer, more relevant information. Finally, the inhibition of irrelevant information occurs in tasks in which students need to inhibit or override the tendency to produce a more dominant or automatic response. In order to make the trained EFs easy to understand and remember, distinctive icons were used to represent them throughout the training program (see Table 2). Adopting an adaptive training perspective, researchers gradually increased the items within each task, as well as the difficulty of the task by increasing, throughout the training sessions, the number of units of information (e.g., words, actions, frames…) to be followed (remembered or integrated), or the distance between critical sentences in the text to answer a comprehension question. Training tasks, examples, variables manipulated for increasing the difficulty, and sessions in which each task was performed are shown in Table 3.

**Table 2:** **The executive processes trained, their icons, and the tasks used in García-Madruga et al. (2013; exp. 2) and Carretti et al. (under revision)**.

**Executive Function**	**Icons**	**Tasks tapping into each executive function**
Focusing	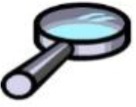	Vignettes in Order, Decoding Instructions, Sentences in Order, Anaphora, Inconsistencies, Inferences, Main Idea, Changing Stories and Integrating Knowledge
Switching	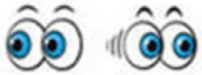	Anaphora, Inconsistencies, Inferences and Integrating Knowledge
Activating and Updating Representations in WM	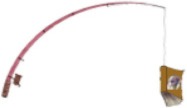	Vignettes in Order, Decoding Instructions, Sentences in Order, Anaphora, Inferences, Main Idea and Changing Stories
	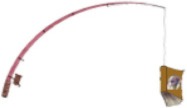	Sentences in Order, Anaphora, Inconsistencies, Inferences, Changing Stories and Integrating Knowledge
Inhibition	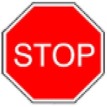	Vignettes in Order, Decoding Instructions, Sentences in Order, Anaphora, Inconsistencies, Main Idea, Changing Stories and Integrating Knowledge

**Table 3:** **Training tasks, examples, variables manipulated for increasing difficulty, sessions in which each task was performed, and the number of items, in García-Madruga et al. (2013, Experiment 2) and Carretti et al. (under revision)**.

**Task**	**Description *Participants were required to…***	**Example of task item**	**Difficulty**	**Sessions**	**Items**
Vignettes in Order	To put in order an increasing number of vignettes	Arrange the following pictures frames	Number of frames	1, 2	50
Decoding written instructions	To read verbal instructions, interpret and perform complex written instructions involving the integration of a sequence of actions	*Write your name and two surnames. Then, draw a circle around the last letter of your name and the first letter of your last surname. Do it without lifting your pencil.*	Number of actions to be performed	2, 3, 4, 5, 6, 7, 8, 9, 10	48
Sentences in Order	To organize series of sentences into the correct order to create a coherent story	Arrange the following sentences: *Maria looks for her place Maria buys the ticket The movie has started Maria waits in the line*	Number of sentences	3, 4	26
Anaphora WM	To solve semantic anaphora, and then store and remember the word solution in a growing series of inferential problems	*Robert painted it white before the summer arrived*. – *roof* – *façade*	Number of words to be remember	4, 5	14
Detecting textual inconsisten-cies	To act as a detective looking for mistakes in a text, either an inconstancy between two ideas expressed or an inconsistency between text and reader's prior knowledge	Internal: *Laura used eyeglasses to read (…) Laura's eyesight was excellent*. External: *Elena was flying in the depths of the lake when he decided to go back.*	*Internal*: distance between sentences *External*: salience of the inconsistency	5, 6, 7	30
Making inferences	To make a text-based inference —integration among individual sentences in the text—, or elaborative inferences —integration of general knowledge with information in the text	(Student reads the text)…Ask the next questions: *Why did they put the sparrow near to the fireplace?*	*Text-based*: Distance between sentences, *Elaborative:* Memory load	6, 7	30
Following changing stories	To read a text including a stream of information in which the relevant facts are constantly changing; to actively keep track of the information as they read it and, at several points of the story, to determine the state of different aspects of the story at that time	*In what order were the horses at the end of the race?*	Number of units of information to be followed	8, 9	18
Integrating information from different formats	To focus and switch attention to different units of information presented on a screen in different formats (i. e., text, video, pictures), in order to be able to answer several questions that required the integration of multiple sources of information	After watching the video and reading the test, ask the following question: *What type of solar eclipse is presented in that picture?*	Number of units of information to be integrated across sources	8, 9	15

The first and last intervention sessions were particularly relevant. In the first session one of the researchers explained in a detailed and direct way the component processes as well as the outcome of reading comprehension. Participants understood and consciously agreed that text comprehension is a real complex task that requires the activation and control of their cognitive resources by using the core EFs. In the last training session students were led to reflect on the utility of the four basic executive processes for diverse daily intellectual activities; likewise, we insisted on the idea that the repeated practice of the four basic processes would be developed such that students could become “mental athletes.” In this final session a personal diploma was presented to each of the students.

Another feature of this training intervention is that it promotes controlled processes through a metacognitive approach, so that the participants receive guidance to recognize and form awareness of the involvement of control processes involved in training program activities, as well as to think about their importance. The instructional techniques used were: (a) explicit instruction by the trainer in the EFs related to the task; (b) modeling examples of the task by the trainer; (c) guided practice; and (d) student independent practice. As a final outcome, the proposal of using repetitive practice was intended to achieve some kind of automated behavior, but always under the control and monitoring of executive processes. That is, it shares features of both implicit and explicit training (Klingberg, 2010).

Our proposed training approach differs in various ways from that of previous training research conducted with children. A key difference is that, instead of intensive training on WM tasks, children performed different text-processing tasks each day selected from the battery of eight tasks included in the training program, as showed in Table 3. Also, unlike many other WM-training studies in which only one training task was used (e.g., Loosli et al., 2012), our training procedure was implemented through a variety of reading comprehension tasks. The tasks we used require increasingly higher attentional control resources and can hence improve students' use of executive processes during reading. Finally, instead of intensive and long training time used in other approaches (e.g., Klingberg et al., 2005), a relatively minimal amount of training time was required in the training program we developed (10–12 sessions of 50 min over 4 weeks).

Evidence for the impact of our training proposal on WM's EFs involved in reading comprehension comes from three studies applying this program to train typical developing children. We expected that even small increases in WM's executive functioning through training would significantly improve children's performance on reading comprehension. These studies attempted to avoid some of the methodological concerns of previous WM training studies (see Shipstead et al., 2012) by using more appropriate WM span tasks, different tasks in the pre- and post-testing than those used in training, more than one measure for WM's EFs and reading comprehension, and active contact groups when possible.

The first study (García-Madruga et al., 2013, exp. 1) was conducted with third-grade students (8–9 years) who were trained for approximately 50 min a day for 12 days over a 4-week period in the classroom. Reading comprehension was assessed at pretest and posttest intervention by means of a Spanish version of the Diagnostic Assessment of Reading Comprehension Test (EDICOLE: August et al., 2006; García-Madruga et al., 2010), a test based on a theoretical analysis of the main components of this ability (Hannon and Daneman, 2001): text information memory, inferences based on information provided in the text, and integration of accessed prior knowledge with new text information. In the experimental group, there was a significant gain after training in the posttest for reading comprehension (Cohen's *d* = 0.67). Moreover, compared with that of a control group that received normal class instruction in Spanish language and reading comprehension, there was a significant higher pretest to posttest gain in the experimental group for reading comprehension, and this effect was large (Cohen's *d* = 0.72). In addition, a Spanish version of the Reading Span Task (RST; Daneman and Carpenter, 1980) for primary school students (Orjales et al., 2010) was used to measure WM capacity at pre- and post-test evaluation. The gain found in favor of the experimental group for RST was not significant. The lack of improvement in this WM measure might be due to the fact that RST is a task that loads mainly on storage and verbal components, even though it is a CE measure.

The second study (García-Madruga et al., 2013, exp 2) was conducted with a larger experimental group and a shorter time period for the entire pretest-intervention-posttest period. Following the procedure described above, the participants (ages 8–9 years) were trained for 10 days in their classroom over a 4-week period. Before and after training, all participants were assessed on reading comprehension by means of the EDICOLE Test, as in Exp. 1, and three complex WM and CE measures of WM capacity. First, a verbal analogy span test for primary-school children (Orjales and García-Madruga, 2010) was used. It has an underlying structure similar to the RST, but instead of only reading aloud and selecting the last word of each sentence, participants have to solve a verbal analogy inference, and store and remember the correct word solution. Second, participants performed a semantic updating span task (Gómez-Veiga et al., 2010; based on Palladino et al., 2001), in which the recall of a variable number of items following a specific semantic criterion in a list of words is measured. Third, a Spanish adaptation of the visuospatial selective span task developed by Cornoldi et al. (2001) was used to assess students' visuospatial WM capacity and the executive processes related to the control of a dual task.

The results of experiment 2 confirmed significant gains after training in the experimental group on the three main components of reading comprehension, and the effects were around medium size: memory and recalling new information presented in the text (Cohen's *d* = 0.33), inferences (Cohen's *d* = 0.62), and integration (Cohen's *d* = 0.65). The effect size for the overall EDICOLE was large (Cohen's *d* = 0.79), as in experiment 1. There was also a significant increase after training on semantic updating and visuospatial WM measures, and the effect was medium to large (Cohen's *d = 0*.62 and 0.77, respectively). However, no significant gain was found after training on the analogy test of WM capacity. As can be noted, the training program yielded greater benefits on the two components of comprehension—inferences and integration—that require an extra mental operation. In these components of reading comprehension, executive control is more involved than it is in text memory. Moreover, the diverse effects of training on participants according to their prior abilities on reading comprehension, as measured by EDICOLE in pretesting, indicate that low reading comprehension students reached a very clear and significant greater gain after training than the high reading comprehension group (Cohen's *d* = 0.34). Since the training program was particularly adapted to the low reading comprehension group, the results support the arguments in favor of adaptive training (e.g., Salminen et al., 2012). Another interesting point is that the use of three tasks in Exp. 2 to evaluate WM training effects, different from those used in training, has allowed us to provide evidence of significant gains in WM's executive functioning and, therefore, a transfer effect of training on the executive process measures. However, the lack of a control group in this experiment requires us to be prudent.

The third study Carretti et al. (under revision) replicated the effects of the training procedure on reading comprehension and extended the results obtained by García-Madruga et al. (2013). The trained group's performance was compared with that of an active and a passive control group before and after the training (10 sessions) and in follow-up sessions 2 months later. The groups were comparable in terms of age (8–9 years old), decoding ability and vocabulary. The active control group took part in standard classroom activities for developing reading comprehension (e.g., read a text and answer different kinds of questions on details of the text). Reading comprehension performance was assessed by using an Italian version of EDICOLE and an Italian standardized reading comprehension test for primary school (Cornoldi and Colpo, 2011). WM capacity was assessed by using an adaptation of the semantic updating span task (Palladino et al., 2001). The results indicated that the trained group—following the procedure described in García-Madruga et al. (2013, exp. 2)—performed better on the standardized measure of comprehension than did both control groups at posttest. At follow up, the trained group performed better than the active or the passive control groups on EDICOLE measures, but the effects were not robust and showed signs of fading. Whereas the trained group's gains from pre- to post-test were medium in terms of effect size for both reading comprehension measures, there was a large effect for WM measure, and the benefits of training were maintained 2 months after intervention.

Overall, the findings regarding the effects of this training approach support the view that it is possible to promote reading comprehension in children by boosting the CE functions during the process of reading, even when training is conducted as part of classroom instruction. Particularly, it provides evidence for the higher contribution of WM's EFs training to those components that require more executive control in reading comprehension tasks: inferences and integration. The results of these studies are consistent with the assumption that cognitive enhancements from our training proposal may have affected not only a specific but also a more domain-general mechanism involved in various executive processes. We note that, since the training tasks took the form of a reading comprehension task and participants were not trained by using WM tasks (except for the anaphora task that share the underlying structure with WSP and the analogy span test), it is difficult to separate the specific differential weight of WM's executive processes training with that of reading comprehension practice to explain the improvement of reading comprehension. Nevertheless, the finding that confirms the efficacy of the intervention in reading comprehension is a relevant result of this training approach. Some transfer effects may also be found by using another complex reading comprehension task to assess the training efficiency. As significant transfer effects were found in different studies (García-Madruga et al., 2013; Carretti et al., under revision), using the same procedure and two different reading comprehension tests that were performed by different experimental groups, we rather think that the beneficial effects found are driven by the training intervention.

As a matter of fact, the training program was relatively brief (10 sessions in Exp. 2), which is shorter than the training time used in other approaches (e.g., Dahlin, 2011), and involved practice distributed over 4 weeks. Even so, this training approach seems to produce effects on measures of WM executive processes and reading comprehension comparable to other training regimes aforementioned, and maintenance effects were also found in reading comprehension as well as in WM measures. The benefits of standardized measures of reading comprehension, as well as those similarly obtained from other training programs, suggests that training improvements may transfer to ecologically valid measures of reading in students of primary school. However, as mentioned, the benefits at follow-up were not robust. Jaeggi et al. (2008) reported dose-dependent effects of training, with more sessions leading to larger transfer effects. Given the reduced number of sessions included in our training program, some further sessions would likely be needed in order to maintain gains at follow up. This hypothesis is requiring, obviously, an empirical test confirmation. In addition, more systematic research is needed to define the optimal intensity and duration of the training intervention.

Finally, we think that training would yield similar results in other complex reading comprehension tasks that demand the precise, deep, and controlled understanding of texts. This hypothesis would require further research and confirmation. In contrast, according our view WM's executive processes training might have smaller impact on those reading tasks that place less demand on WM such as basic word reading skills (see Holmes et al., 2009; Dahlin, 2011; Loosli et al., 2012).

## Conclusions

In the current paper we have presented an overall view of EFs that includes three main kinds of processes: core on-line EFs, higher order EFs, and emotional control. This theoretical proposal is based in recent theoretical and experimental breakthroughs and attempts to clarify the relations between WM, EFs, and higher-level cognition. A corollary of this theory is that we can improve thinking abilities by improving the use of executive functions during the process of solving complex cognitive tasks involved in each kind of thinking ability. We have also presented an instructional program to improve reading comprehension based on training the core executive processes involved in the solution to a set of selected tasks, as well as the main results found in a set of training experiments recently carried out.

As briefly discussed, an important feature of executive processes is that they are potentially modifiable. There is a considerable amount of evidence on WM and executive function interventions and training, although their overall generalizing effect is still a matter of debate. Our proposal does not require a high generalizing effect since we are intervening on the executive processes involved in a set of tasks that represent the complexities and difficulties of reading comprehension. Our experimental findings on reading comprehension confirm an improvement in reading comprehension and WM executive processing measures in posttest and follow-up measures. From our theoretical perspective we certainly expect some kind of generalization. Given that the same EFs are involved in two different higher-level cognitive abilities, generalizing the results between the two abilities is possible. Therefore, we share the idea of a domain-general mechanism (WM and EFs) that might underlie the generalization effect found recently in diverse studies (Klingberg et al., 2005; Buschkuehl et al., 2008; Jaeggi et al., 2008; Persson and Reuter-Lorenz, 2008; Chein and Morrison, 2010). However, our view maintains that if we want to achieve robust and significant effects, the repeated and adaptive training of complex WM task is not enough. Instead, we have to go a step further and train the WM's executive functions involved in solving a set of representative tasks of the particular higher-level cognitive ability we want to improve. In other words, our view maintains that in order to improve thinking abilities the domain-general mechanism is insufficient. There are also domain-specific competences requiring the active use of EFs which have also to be trained.

Moreover, higher-level cognitive abilities cannot be reduced to the EFs involved in them. In other words, we do not agree with Diamond when she explicitly says that higher-level thinking, reasoning and creativity are in fact EFs. In our opinion, these higher-level intellectual abilities, that is, thinking and fluid intelligence, share the crucial role of EFs, but they are themselves not EFs. They are the result of applying executive processes to solve particular kinds of intellectual problems. They entail the manipulation of diverse kinds of representations, use diverse beginning and ending points, follow different thinking sequences, and have diverse aims.

Our view on the relationship between higher-level abilities and EFs suggests that a similar improvement to that obtained in reading comprehension can be achieved in other higher-level abilities as reasoning and problem solving. The design and development of the instructional programs for improving other intellectual abilities entails two main components: (a) a general theoretical view on EFs and how they can be instructed; and (b) a specific theoretical analysis of each of the higher-level abilities and the role of the EFs that operate on it, that will allow an adequate selection of the tasks to be instructed. A new training program on WM's executive functions to improve deductive reasoning in Secondary school students has been designed by our research group (García-Madruga et al., 2015) but not yet experimentally tested. Like reading comprehension, deductive reasoning requires the construction of representations, but its peculiar feature is to manipulate these representations in order to arrive at, if possible, a necessary conclusion. This goal-oriented sequential task of manipulating representations is performed in WM and can be defined as a kind of updating process driven by reasoners' meta-deductive knowledge and goals. The program is based on training participants in the four core EFs and a higher order one: revision. According to our view, these EFs underlie the application to solving diverse deductive tasks of two meta-deductive concepts (consistency and necessity) and two meta-deductive strategies (searching for counterexamples and exhaustivity).

Finally, we would like to address various limitations and perspectives of our theoretical conception regarding the improvement of thinking abilities. We have outlined the preliminary character of our theoretical view on EFs. Our proposal still requires empirical verification, particularly the two cognitive higher-order EFs, planning and revision, and their relationship with WM and core EFs, as well as their developmental pattern. Our experimental work has tested the efficacy of a program to improve reading comprehension, but not our theoretical view on EFs. In this regard, a second main limitation affects our experimental work. Given the overlapping nature of the core EFs involved in reading comprehension, our training experiments do not allow us to differentiate the role of each of the four core EFs. It is possible however to evaluate the relevance of each of the tasks used in training, as in fact is done in our second experiment (García-Madruga et al., 2013).

We are now working on an obvious testable prediction of our view: the empirical comparison of training efficacy between our instructional program on WM's executive functions involved in reading comprehension and an equivalent program based only on training the verbal and spatial complex WM task used by Chein and Morrison (2010), the n-back WM task frequently used in training studies, and our Analogy and Anaphora WM tasks. For that purpose, we will use diverse pre- and post-training measures of reading comprehension, WM's executive processes and fluid intelligence. Likewise, a more detailed analysis and evaluation might be done in the future with respect to the impact of higher order cognitive EFs such as planning and revision. For instance, the specific role of planning and revision in problem solving and reasoning, respectively, might be evaluated by introducing (or not) the training of these EFs, other than the core EFs, in instructional programs to improve these intellectual abilities.

The final aim of our programs to improve reading comprehension or reasoning through the intervention on the relevant EFs also includes moving beyond these abilities in our attempt to improve education. EFs are involved in every learning task that requires a cognitively active and controlled performance from the learner (see e.g., Meltzer, 2007). The role of EFs is therefore crucial in the acquisition of basic instrumental skills such as reading, writing, and arithmetic. Likewise, EFs are required in the acquisition of diverse kinds of academic content. For instance, complex declarative learning is another higher-level intellectual ability in which EFs are obviously involved and one that directly depends on reading comprehension and reasoning. In a directly related way, WM and EFs deficits underlie learning and intellectual disability. The intervention on the EFs particularly involved in diverse learning and intellectual disabilities is thus a promising way to improve an individual's performance. According to our view, these interventions demand a previous and detailed analysis of each particular disability and the role of EFs involved in it, in order to design an instructional program and select the appropriate training tasks. Although WM and EFs play a role as a domain-general mechanism that underlies intellectual and learning disabilities, there is not a domain-general procedure of improving them. Domain-specific procedures and programs to improve WM and EFs are required if we want to improve individuals' learning and intellectual disabilities.

## Author contributions

All authors listed, have made substantial, direct and intellectual contribution to the work, and approved it for publication.

### Conflict of interest statement

The authors declare that the research was conducted in the absence of any commercial or financial relationships that could be construed as a potential conflict of interest. The reviewer, Maja Roch, and handling Editor declared their shared affiliation, and the handling Editor states that the process nevertheless met the standards of a fair and objective review.

## References

[B1] Agostino A. Johnson J. Pascual-Leone J. (2010). Executive functions underlying multiplicative reasoning: problem type matters. J. Exp. Child Psychol. 105, 286–305. 10.1016/j.jecp.2009.09.006 19913238

[B2] Aker M. Landro N. I. (2014). Executive control of emotional processing: a set-shifting task. Clin. Neuropsychol. 28, 1211–1220. 10.1080/13854046.2014.984762 25422031

[B3] Allal L. Chanquoy L. Largy P. (2004). Revision Cognitive and Instructional Processes. New York, NY: Kluwer Academic Publishers.

[B4] Alvarez J. A. Emory E. (2004). Executive function and the frontal lobes: a metaanalytic review. Neuropsychol. Rev. 16, 17–42. 10.1007/s11065-006-9002-x 16794878

[B5] August D. Francis D. J. Hsu H. A. Snow C. E. (2006). Assessing reading comprehension in bilinguals. Elem. Sch. J. 107, 221–238. 10.1086/510656

[B6] Awh E. Vogel E. K. Oh S. (2006). Interactions between attention and working memory. Neuroscience 139, 201–208. 10.1016/j.neuroscience.2005.08.023 16324792

[B7] Baddeley A. D. (1986). Working Memory. Oxford, UK: Oxford University Press.

[B8] Baddeley A. D. (2000). The episodic buffer: a new component of working memory? Trends Cognit. Sci. 4, 417–423. 10.1016/S1364-6613(00)01538-2 11058819

[B9] Baddeley A. D. (2007). Working Memory, Thought, and Action. Oxford, UK: Oxford University Press.

[B10] Baddeley A. D. Hitch G. (1974). Working memory, in The Psychology of Learning and Motivation, Vol. 8, ed Bower G.A. (New York, NY: Academic), 47–89.

[B11] Belachi C. Carretti B. Cornoldi C. (2010). The role of working memory and updating in Coloured Raven Matrices performance in typically developing children. Eur. J. Cogn. Psychol. 22, 1010–1020. 10.1080/09541440903184617

[B12] Best J. R. Miller P. H. Jones L. L. (2009). Executive function after age 5: changes and correlates. Dev. Rev. 29, 180–200. 10.1016/j.dr.2009.05.002 20161467 PMC2792574

[B13] Blakemore S. Frith U. (2005). Learning Brain. Oxford, UK: Blackwell

[B14] Borella E. Carretti B. De Beni R. (2008). Working memory and inhibition across the adult life-span. Acta Psychol. 128, 33–44. 10.1016/j.actpsy.2007.09.008 17983608

[B15] Borella E. Carretti B. Riboldi F. De Beni R. (2010). Working memory training in older adults: evidence of transfer and maintenance effects. Psychol. Aging 25, 767–778. 10.1037/a0020683 20973604

[B16] Broadbent D. E. (1958). Perception and Communication. New York, NY: Oxford University Press.

[B17] Buschkuehl M. Jaeggi S. Kobel A. Perrig W. J. (2008). Brain Twister Aufgabensammlung für Kognitives Training. Bern: Universitat Bern; Institut fur Psychologie.

[B18] Cain K. Oakhill J. Bryant P. (2004). Children's reading comprehension ability: concurrent prediction by working memory, verbal ability, and component skills. J. Educ. Psychol. 96, 31–42. 10.1037/0022-0663.96.1.31

[B19] Carretti B. Borella E. Cornoldi C. De Beni R. (2009). Role of working memory in explaining the performance of individuals with specific reading comprehension difficulties: a meta-analysis. Learn. Individ. Diff. 19, 246–251. 10.1016/j.lindif.2008.10.002

[B20] Carretti B. Caldarola N. Tencati C. Cornoldi C. (2014). Improving reading comprehension in reading and listening settings: the effect of two training programmes focusing on metacognition and working memory. Br. J. Educ. Psychol. 84, 194–210. 10.1111/bjep.12022 24829118

[B21] Carretti B. Cornoldi C. De Beni R. Romanó M. (2005). Updating in working memory: a comparison of poor and good comprehenders. J. Exp. Child Psychol. 91, 45–66. 10.1016/j.jecp.2005.01.005 15814095

[B22] Chein J. Morrison A. (2010). Expanding the mind's workspace: training and transfer effects with a complex working memory span task. Psychon. Bull. Rev. 17, 193–199. 10.3758/PBR.17.2.193 20382919

[B23] Chen T. Li D. (2007). The roles of working memory updating and processing speed in mediating age-related differences in fluid intelligence. Aging Neuropsychol. Cogn. 14, 631–646. 10.1080/13825580600987660 18038360

[B24] Collette F. Van der Linden M. Laureys S. Delfiore G. Degueldre C. Luxen A.. (2005). Exploring the unity and diversity of the neural substrates of executive functioning. Hum. Brain Mapp. 25, 409–423. 10.1002/hbm.20118 15852470 PMC6871684

[B25] Collins A. Koechlin E. (2012). Reasoning, learning, and creativity: frontal lobe function and human decisión making. PLoS Biol. 10:e1001293. 10.1371/journal.pbio.1001293 22479152 PMC3313946

[B26] Cornoldi C. Colpo G. (2011). Prove di lettura MT. Aggiornamento 2011. Firenze: Organizzazionni Speciali.

[B27] Cornoldi C. Marzocchi G. M. Belotti M. Caroli M. G. Meo T. Braga C. (2001). Working memory interference control deficit in children referred by teachers for ADHD symptoms. Child Neurospychol. 7, 230–240. 10.1076/chin.7.4.230.8735 16210212

[B28] Cowan N. (1999). An embedded-processes model of working memory, in Models of Working Memory: Mechanisms of Active Maintenance and Executive Control, eds Miyakey A. Shah P. (Cambridge: Cambridge University Press), 62–101.

[B29] Cowan N. (2005). Working Memory Capacity. Nueva York, NY: Psychology Press.

[B30] Cummings J. L. (1993). Frontal subcortical circuits and human behaviour. Arch. Neuro. 50, 873–880. 10.1001/archneur.1993.00540080076020 8352676

[B31] Dahlin K. I. E. (2011). Effects of working memory training on reading in children with special needs. Read. Writ. 24, 479–491. 10.1007/s11145-010-9238-y

[B32] Daneman M. Carpenter P. (1980). Individual differences in working memory and reading. J. Verbal Learn. Verbal Behav. 19, 450–466. 10.1016/S0022-5371(80)90312-6

[B33] De Beni R. Palladino P. (2000). Intrusion errors in working memory tasks. Are they related with reading comprehension ability? Learn. Individ. Diff. 12, 131–143. 10.1016/S1041-6080(01)00033-4

[B34] De Jong P. F. (2006). Understanding normal and impaired reading development: a working memory perspective, in Working Memory and Education, ed Pickering S. (Amsterdam: Elsevier), 33–60.

[B35] Diamond A. (2002). Normal development of prefrontal cortex from birth to young adulthood: cognitive functions, anatomy, and biochemistry, in Principles of Frontal Lobe Function, eds Stuss D. T. Knight R. T. (London: Oxford University Press), 466–503.

[B36] Diamond A. (2006). The early development of executive functions, in Lifespan Cognition: Mechanisms of Change, eds Bialystok E. Craik F. (New York, NY: Oxford University Press), 70–95.

[B37] Diamond A. (2009). All or none hypothesis: a global-default mode that characterizes the brain and mind. Dev. Psychol. 45, 130–138. 10.1037/a0014025 19209996 PMC2643366

[B38] Diamond A. (2012). Activities and programs that improve children's executive functions. Curr. Dir. Psychol. Sci. 21, 335–341. 10.1177/0963721412453722 25328287 PMC4200392

[B39] Diamond A. (2013). Executive Functions. Annu. Rev. Psychol. 64, 135–168. 10.1146/annurev-psych-113011-143750 23020641 PMC4084861

[B40] Dunning D. L. Holmes J. (2014). Does working memory training promote the use of strategies on untrained working memory tasks? Mem. Cognit. 42, 854–862. 10.3758/s13421-014-0410-5 24748348 PMC4110412

[B41] Engle R. W. (2001). What is working memory capacity, in The Nature of Remembering: Essays in Honor of Robert G. Crowder, eds Roediger H. L. Naime J. S. Neath I. Supremant A. M. (Washington, DC: American Psychological Association), 297–314.

[B42] Engle R. W. (2002). Working memory capacity as executive attention. Curr. Dir. Psychol. Sci. 11, 19–23. 10.1111/1467-8721.00160

[B43] Ericsson K. A. Kintsch W. (1995). Long-term working memory. Psychol. Rev. 102, 211–245. 10.1037/0033-295X.102.2.211 7740089

[B44] Frederick S. (2005). Cognitive reflection and decision making. J. Econ. Perspect. 19, 25–42. 10.1257/089533005775196732

[B45] Friedman N. P. Miyake A. Corley R. P. Young S. E. DeFries J. C. Hewitt J. K. (2006). Not all executive functions are related to intelligence. Psychol. Sci. 17, 172–179. 10.1111/j.1467-9280.2006.01681.x 16466426

[B46] Fuster J. M. (1989). The Prefrontal Cortex: Anatomy, Physiology and Neuropsychology of the Frontal Lobe, 2nd Edn New York, NY: Raven Press.

[B47] García-Madruga J. A. Elosúa M. R. Gil L. Gómez-Veiga I. Vila J. Ó. Orjales I. (2013). Reading comprehension and working memory's executive processes: an intervention study in primary school students. Read. Res. Quart. 48, 155–174. 10.1002/rrq.44

[B48] García-Madruga J. A. Gómez-Veiga I. Vila J. Ó. (2015). A Program of Intervention on Executive Functions to Improve Deductive Reasoning. Pesaro: Póster presented at XXIV Congresso Nazionale Airipa.

[B49] García Madruga J. A. Pérez E. Gómez-Veiga I. Orjales I. Gil L. Elosúa R. (2010). Prueba de comprensión lectora para ense1anza primaria: EDICOLE (evaluación diagnóstica de la comprensión lectora). Unpublished work.

[B51] Garon N. Bryson S. E. Smith I. M. (2008). Executive function in preschoolers: a review using an integrative framework. Psychol. Bull. 134, 31–60. 10.1037/0033-2909.134.1.31 18193994

[B52] Gaskins I. W. Satlow E. Pressley M. (2007). Executive control of reading comprehension in the elementary school, in Executive Function in Education: From Theory to Practice, ed Meltzer L. (New York, NY: Guilford Press), 194–215.

[B53] Gathercole S. E. Alloway T. P. (2008). Working Memory and Learning: A Practical Guide. London: Sage Press.

[B54] Gathercole S. E. Lamont E. Alloway T. P. (2006). Working memory in the classroom, in Working Memory and Education, ed Pickering S. (San Diego, CA: Academic Press), 219–240.

[B55] Gazzaley A. Nobre A. C. (2011). Top-down modulation: bridging selective attention and working memory. Trends Cognit. Sci. 16, 129–135. 10.1016/j.tics.2011.11.014 22209601 PMC3510782

[B56] Goel V. Grafman J. (1995). Are the frontal lobes implicated in “planning” functions? Interpreting data from the Tower of Hanoi. Neuropsychologia 33, 623–642. 10.1016/0028-3932(95)90866-P 7637857

[B50] Gómez-Veiga I. Contreras A. García-Madruga J. A. (2010). Prueba de Actualización Semántica. PASE-Primaria. Unpublished work.

[B57] Hannon B. Daneman M. (2001). A new tool for measuring and understanding individual differences in the component processes of reading comprehension. J. Educ. Psychol. 93, 103–128. 10.1037/0022-0663.93.1.103

[B58] Holmes J. Gathercole S. E. (2014). Taking working memory training from the laboratory into schools. Educ. Psychol. 34, 1–11. 10.1080/01443410.2013.797338 26494933 PMC4579053

[B59] Holmes J. Gathercole S. E. Dunning D. L. (2009). Adaptive training leads to sustained enhancement of poor working memory in children. Dev. Sci. 12, F9–F15. 10.1111/j.1467-7687.2009.00848.x 19635074

[B60] Huizinga M. Dolan C. V. van der Molen M. W. (2006). Age-related change in executive function: developmental trends and a latent variable analysis. Neuropsychologia 44, 2017–2036. 10.1016/j.neuropsychologia.2006.01.010 16527316

[B61] Im-Bolter N. Johnson J. Pascual-Leone J. (2006). processing limitations in children with specific language impairment: the role of executive function. Child Dev. 77, 1822–1841. 10.1111/j.1467-8624.2006.00976.x 17107463

[B62] Jaeggi S. M. Buschkuehl M. Jonides J. Perrig W. J. (2008). Improving fluid intelligence with training on working memory. Proc. Natl. Acad. Sci. U.S.A. 105, 6829–6833. 10.1073/pnas.0801268105 18443283 PMC2383929

[B63] Jaeggi S. M. Buschkuehl M. Jonides J. Shah P. (2011). Short- and long-term benefits of cognitive training. Proc. Natl. Acad. Sci. U.S.A. 108, 10081–10086. 10.1073/pnas.1103228108 21670271 PMC3121868

[B64] Johnson M. H. (1998). The neural basis of cognitive development, in Handbook of Child Psycholog: Vol. 2, Cognition Perception, and Language eds Damon W. Kuhn D. Siegler R. S. (New York, NY: Wiley), 1–50.

[B65] Jolles D. D. Kleibeuker S. W. Rombouts S. A. R. B. Crone E. A. (2011). Developmental differences in prefrontal activation during working memory maintenance and manipulation for different memory loads. Dev. Sci. 14, 731–724. 10.1111/j.1467-7687.2010.01016.x 21676092

[B66] Jurado M. B. Rosselli M. (2007). The elusive nature of executive functions: a review of our current understanding. Neuropsychol. Rev. 17, 213–233. 10.1007/s11065-007-9040-z 17786559

[B67] Just M. A. Carpenter P. A. (1992). A capacity theory of comprehension. Psychol. Rev. 99, 122–149. 10.1037/0033-295X.99.1.122 1546114

[B68] Karbach J. Kray J. (2009). How useful is executive control training. Age differences in near and far transfer of task-switching training. Dev. Sci. 12, 978–990. 10.1111/j.1467-7687.2009.00846.x 19840052

[B69] Karbach J. Schubert T. (2013). Training-induced cognitive and neural plasticity. Front. Hum. Neurosci. 7:48. 10.3389/fnhum.2013.00048 23437015 PMC3579194

[B70] Karbach J. Strobach T. Schubert T. (2015). Adaptive working-memory training benefits reading, but not mathematics in middle childhood. Child Neuropsychol. 21, 285–301. 10.1080/09297049.2014.899336 24697256

[B71] Karbach J. Unger K. (2014). Executive control training from middle childhood to adolescence. Front. Psychol. 5:390. 10.3389/fpsyg.2014.00390 24847294 PMC4019883

[B72] Kintsch W. (1998). Comprehension. A Paradigm for Cognition. Cambridge, MA: Cambridge University Press.

[B73] Klenberg L. Korkman M. Lahti-Nuuttila P. (2001). Differential development of attention and executive functions in 3- to 12-year-old finnish children. Dev. Neuropsychol. 20, 407–428. 10.1207/S15326942DN2001_6 11827096

[B74] Klingberg T. (2010). Training and plasticity of working memory. Trends Cognit. Sci. 13, 317–324. 10.1016/j.tics.2010.05.002 20630350

[B75] Klingberg T. Fernell E. Olesen P. J. Johnson M. Gustafsson P. Dahlstrom K.. (2005). Computerized training of workingmemory in children with ADHD–a randomized, controlled trial. J. Am. Acad. Child Adolesc. Psychiatry 44, 177–186. 10.1097/00004583-200502000-00010 15689731

[B76] Klingberg T. Forssberg H. Westerberg H. (2002). Training of working memory in children with ADHD. J. Clin. Exp. Neuropsychol. 24, 781–791. 10.1076/jcen.24.6.781.8395 12424652

[B77] Lezak M. D. (1982). The problema of assessing exectuive functions. Int. J. Psychol. 17, 281–297. 10.1080/00207598208247445

[B78] Loosli S. V. Buschkuehl M. Perrig W. J. Jaeggi S. M. (2012). Working memory training improves reading processes in typically developing children. Child Neuropsychol. 18, 62–78. 10.1080/09297049.2011.575772 21623483

[B79] Lunt L. Bramham J. Morris R. G. Bullock P. R. Selway R. P.. (2012). Prefrontal cortex dysfunction and “jumping to conclusions”: bias or deficit? J. Neuropsychol. 6, 65–78. 10.1111/j.1748-6653.2011.02005.x 22257612

[B80] Luria A. R. (1966). Human Brain and Psychological Processes. New York, NY: Harper and Row.

[B81] Melby-Lervâg M. Hulme C. (2013). Is working memory training effective? A meta-analytic review. Dev. Psychol. 49, 270–291. 10.1037/a0028228 22612437

[B82] Meltzer L. J. (2007). Executive Function in Education: From theory to Practice. New York, NY: Guilford Press.

[B83] Meltzer L. Pollica L. Barzillai M. (2007). Executive function in the classroom: embedding strategy instruction into daily teaching practices, in Executive Function in Education: From Theory to Practice, ed Meltzer L. (New York, NY: Guilford Press), 165–193.

[B84] Miyake A. Friedman N. P. (2012). The nature and organization of individual differences in executive functions: four general conclusions. Curr. Dir. Psychol. Sci. 21, 8–14. 10.1177/0963721411429458 22773897 PMC3388901

[B85] Miyake A. Friedman N. P. Emerson M. J. Witzki A. H. Howerter A. Wagner T. D. (2000). The unity and diversity of executive functions and their contributions to complex “frontal lobe” tasks: a latent variable analysis. Cogn. Psychol. 41, 49–100. 10.1006/cogp.1999.0734 10945922

[B86] Morris N. Jones D. M. (1990). Memory updating in working memory: the role of the central executive. Br. J. Psychol. 81, 111–121. 10.1111/j.2044-8295.1990.tb02349.x

[B87] Morris R. Ward G. (2005). The Cognitive Psychology of Planning. Hove: Psychology Press

[B88] Morrison A. Chein J. (2011). Does working memory training work? The promise and challenges of enhancing cognition by training working memory. Psychon. Bull. Rev. 18, 46–60. 10.3758/s13423-010-0034-0 21327348

[B89] Nelson C. A. Thomas K. M. de Haan M. (2006). Neural bases of cognitive development,. In Handbook of Child Psychology, 6th Edn., Vol. 2, Cognitive Development, eds Kuhn D. Siegler R. (Hoboken, NJ: Wiley and Sons), 3–48.

[B90] Nobre A. C. Stokes M. G. (2011). Attention and short-term memory: crossroads. Neuropsychologia 49, 1391–1392. 10.1016/j.neuropsychologia.2011.04.014 21571124

[B91] Norman D. A. Shallice T. (1986). Attention to action: willed and automatic control of behavior, in Consciousness and Self-Regulation: Advances in Research and Theory, Vol. 4, eds Davidson R. J. Schwartz G. E. Shapiro D. (New York, NY: Plenum Press), 1–18.

[B92] Nurmi J.-E. (1991). How do adolescents see their future? A review of the development of future orientation and planning. Dev. Rev. 11, 1–59

[B93] Orjales I. García-Madruga J. A. (2010). Prueba de Analogías Para Primaria (Analogies Span Test for Primary School). Unpublished manuscript.

[B94] Orjales I. García-Madruga J. A. Elosúa M. R. (2010). Prueba de Amplitud Lectora Para Primaria [Reading Span Test for Primary School]. Unpublished manuscript.

[B95] Palladino P. Cornoldi C. De Beni R. Pazzaglia F. (2001). Working memory and updating processes in reading comprehension. Mem. Cognit. 29, 344–354. 10.3758/BF03194929 11352218

[B96] Pascual-Leone J. (1987). Organismic processes for neo-Piagetian theories: a dialectical causal account of cognitive development. Int. J. Psychol. 22, 531–570. 10.1080/00207598708246795

[B97] Pascual-Leone J. (2000). Reflections on working memory: are the two models complementary? J. Exp. Child Psychol. 77, 138–154. 10.1006/jecp.2000.2593 11017722

[B98] Persson J. Reuter-Lorenz P. A. (2008). Gaining control: training of executive function and far transfer of the ability to resolve interference. Psychol. Sci. 19, 881–889. 10.1111/j.1467-9280.2008.02172.x 18947353

[B99] Posner M. I. (1980). Orienting of attention. Q. J. Exp. Psychol. 32, 3–25. 10.1080/00335558008248231 7367577

[B100] Posner M. I. Sheese B. E. Odludas Y. Tong Y. (2006). Analyzing and shaping human attentional networks. Neural Netw. 19, 1422–1429. 10.1016/j.neunet.2006.08.004 17059879

[B101] Rothbart M. K. Posner M. I. (2001). Mechanism and variation in the development of attentional networks, in Handbook of Developmental Cognitive Neuroscience, eds Nelson C. A. Luciana M. (Cambridge, MA: MIT Press), 353–363.

[B102] Salminen T. Strobach T. Schubert T. (2012). On the impacts of working memory training on executive functioning. Front. Hum. Neurosci. 6:166 10.3389/fnhum.2012.00166 PMC3368385 22685428

[B103] Shiffrin R. M. Schneider W. (1977). Controlled and automatic human information processing. II. Perceptual learning, automatic attending and a general theory. Psycholo Rev. 84, 127–190. 10.1037/0033-295X.84.2.127

[B104] Shipstead Z. Redick T. W. Engle R. W. (2012). Is working memory training effective? Psychol. Bull. 138, 628–654. 10.1037/a0027473 22409508

[B105] Swanson H. Alloway T. (2012). Working memory, learning, and academic achievement, in Educational Psychology Handbood, Vol. 1: Theories, Constructs, and Critical Issues, eds Harris K. R. Graham S. Urdan T. C. McCormick Chritine B. G. Sinatra M. Sweller J. (Washington, DC: APA), 327–366.

[B106] Swanson H. L. Howard C. Saez L. (2006). Components of working memory that are related to poor reading comprehension and word recognition performance in less skilled readers. J. Learn. Disabil. 39, 252–269. 16724796 10.1177/00222194060390030501

[B107] St. Clair-Thompson H. Stevens R. Hunt A. Bolder E. (2010). Improving children's working memory and classroom performance. Educ. Psychol. Int. J. Exp. Educ. Psychol. 30, 203–219. 10.1080/01443410903509259

[B108] Titz C. Karbach J. (2014). Working Memory and executive functions: effects of training on academic achievement. Psychol. Res. 78, 852–868. 10.1007/s00426-013-0537-1 24389706

[B109] Unsworth N. Engle R. W. (2007). The nature of individual differences in working memory capacity: active maintenance in primary memory and controlled search from secondary memory. Psychol. Rev. 114, 104–132. 10.1037/0033-295X.114.1.104 17227183

[B110] von Bastian C. C. Oberauer K. (2014). Effects and mechanisms of working memory training: a review. Psychol. Res. 78, 803–820. 10.1007/s00426-013-0524-6 24213250

[B111] Wager T. Smith E. E. (2003). Neuroimaging studies of working memory: a meta-analysis. Cognit. Affect. Behav. Neurosci. 3, 255–274. 10.3758/CABN.3.4.255 15040547

[B112] Yeniad N. Malda M. Mesman J. van Ijzendoorn M. H. Pieper S. (2013). Shifting ability predicts math and reading performance in children: a meta-analytical study. Learn. Individ. Diff. 23, 1–9. 10.1016/j.lindif.2012.10.004

